# Metabolic Investigation in *Gluconacetobacter xylinus* and Its Bacterial Cellulose Production under a Direct Current Electric Field

**DOI:** 10.3389/fmicb.2016.00331

**Published:** 2016-03-17

**Authors:** Miao Liu, Cheng Zhong, Yu Ming Zhang, Ze Ming Xu, Chang Sheng Qiao, Shi Ru Jia

**Affiliations:** ^1^Key Laboratory of Industrial Fermentation Microbiology, Ministry of Education, Tianjin University of Science and TechnologyTianjin, China; ^2^Key Laboratory of Systems Bioengineering, Ministry of Education, Tianjin UniversityTianjin, China

**Keywords:** bacterial cellulose, *Gluconacetobacter xylinus*, metabolic profiles, lactic acid, water electrolysis

## Abstract

The effects of a direct current (DC) electric field on the growth and metabolism of *Gluconacetobacter xylinus* were investigated in static culture. When a DC electric field at 10 mA was applied using platinum electrodes to the culture broth, bacterial cellulose (BC) production was promoted in 12 h but was inhibited in the last 12 h as compared to the control (without DC electric field). At the cathode, the presence of the hydrogen generated a strong reductive environment that is beneficial to cell growth. As compared to the control, the activities of glycolysis and tricarboxylic acid cycle, as well as BC productivity were observed to be slightly higher in the first 12 h. However, due to the absence of sufficient oxygen, lactic acid was accumulated from pyruvic acid at 18 h, which was not in favor of BC production. At the anode, DC inhibited cell growth in 6 h when compared to the control. The metabolic activity in *G. xylinus* was inhibited through the suppression of the tricarboxylic acid cycle and glycolysis. At 18–24 h, cell density was observed to decrease, which might be due to the electrolysis of water that significantly dropped the pH of cultural broth far beyond the optimal range. Meanwhile, metabolites for self-protection were accumulated, for instance proline, glutamic acid, gluconic acid, and fatty acids. Notably, the accumulation of gluconic acid and lactic acid made it a really tough acid stress to cells at the anode and finally led to depression of cell growth.

## Introduction

Cellulose synthesized by bacteria is called bacterial cellulose (BC) (Brown et al., 1976). BC synthesis is a complex process: the polymerization of single glucose molecules into β-1,4-glucan chains; the extracellular “extrusion” of the linear polymer chains; and the assembly of the chains into ribbon-like fibrils (nanometer scale) (Brown et al., 1982). BC exhibits a fine three-dimensional random network, which has attracted an increasing interest due to its high quality in purity, biocompatibility, mechanical integrity, and its stability (Brown et al., 1982). Till now, BC has been successfully applied in several biomedical applications including blood vessel replacement, meniscus, and bone scaffolds (Kwak et al., 2015). However, current fabrication techniques suffer from several manufacturing challenges in the control of shape and size that have, to date, inhibited their clinical applications. These limitations result from an inability to reproducibly create BC structures on the nano-, micro-, and millimeter scales that adequately promote growth and function of implanted cells.

Researchers have made endeavors toward overcoming these limitations. Bodin (Bodin et al., 2007) manipulated the shape and morphology of BC by delivering oxygen to the culture media through a polymeric interface. Putra (Putra et al., 2008) found that BC tubes grown on silicone tubing showed nanofibers orientation along the longitudinal axis. Kondo (Kondo et al., 2002) got epitaxial-aligned BC nanofibers on a molecular substrate of aligned glucan chains. Besides, the network structure of BC might be altered by bacterial action which could be affected by chemical ingredients, pH, temperature, electric or magnetic forces, etc. (Zareie et al., 2003; Sano et al., 2010). Baah-Dwomoh (Baah-Dwomoh et al., 2015) demonstrated the feasibility of using irreversible electroporation to introduce pores in BC by killing most of the bacteria. In our previous studies (Yan et al., 2012; Zheng et al., 2014), electric field was used to control motion of *Gluconacetobacter xylinus* (Yamada et al., 2012), which allowed for different orientations and organization of the cellulose fibers. This provided a means of fabricating customizable networks and could in turn modulate the mechanical properties of BC. The results were consistent with a previous study (Sano et al., 2010). It suggested that similar techniques could be used to create BC materials with distinct properties to meet the applications in different fields. However, there are still some problems to be solved with regard to electric field, such as the low BC yield and the high cell fatality rate. More attentions should be paid to the mechanism how electric field affected bacterial cells. However, till now, very few reports are available in this field.

The present study is aimed to get insight into the metabolism of *G. xylinus* CGMCC 2955 as well as BC production either in the presence or absence of electric field. The direct current (DC) electric field was performed at the current intensity of 10 mA. Instead of focusing on the overall effect, we revealed the cellular metabolomics and BC production in the anode and cathode, respectively. Intra-cellular metabolic differences were profiled by gas chromatography-mass spectrometer (GC-MS) based metabolomics approach coupled with multivariate statistical analysis. The metabolic response of *G. xylinus* CGMCC 2955 to DC electric field and its correlation with BC production were elucidated.

## Materials and methods

### Microorganisms and culture media

*G. xylinus* CGMCC 2955 was isolated from 16 solid fermentation substrates of vinegar in the Key Laboratory of Industrial Fermentation Microbiology, Tianjin University of Science and Technology, and it was deposited in China General Microbiological Center Collection (CGMCC) with the registered number 2955. Sequencing of the 16S rRNA gene and the construction of a phylogenetic tree were carried out as reported previously (Yamada et al., 2000). The 16S rDNA gene was amplified by PCR with two primers: 27F (5′- AGAGTTTGATCCTGGCTCTAG -3′), 1492R (5′- GGTTACTTGTTACGACTT -3′). The purified PCR products were sequenced directly by Genewiz. Inc. A phylogenetic tree based on 16S rDNA gene sequences of type strains of the species of the family *Gluconacetobacter* was constructed with the MEGA 5.1. Neighbor-joining tree was estimated by bootstrapping with 1000 replicates.

The culture media contained (g/L): glucose 25.0, peptone 10.0, yeast extract 7.5, and disodium phosphate 10.0, with an initial pH of 6.0.

### Pre-culture and DC treatment experiments

*G. xylinus* CGMCC 2955 was cultured as previously reported (Zhong et al., 2013). The initial cell density of inoculum was adjusted to 0.5 (OD_600_), and cells were then inoculated to 500 mL of fresh culture medium at a ratio of 6.0% (v/v) into two identical vessels, respectively (Beijing LIUYI Biotechnology Co., LTD, China). As shown in Figure S1, the DC treatment experiments were conducted in the two vessels inserted with two platinum (Pt) electrodes (ϕ = 0.2 mm, L = 115.0 mm). Prior to the DC treatment, cells were pre-cultured for 24 h to the early exponential phase (0.3 in OD_600_). A DC electric field at the current intensity of 10 mA held constant was then applied to one vessel, and another identical vessel was used as a control. The temperature was maintained at 30°C for 24 h. Samples were taken periodically (every 6 h) from each vessel and immediately analyzed.

### Membrane-disrupting activity (PI assay)

The membrane-disrupting activity of DC treatment on *G. xylinus* was determined by measuring the fluorescence enhancement of prodium iodide (PI, Sigma) according to a modified method (Shafiei et al., 2013). PI only enters cells whose membrane was damaged, after which the fluorescence in cells can be enhanced by 20–30 folds due to its binding to nucleic acids. *G. xylinus* cells were harvested by centrifugation at 4000 rpm for 5 min, and washed with phosphate buffer solution (PBS) twice. The washed pellets were resuspended in PBS to a final cell density of 0.3–0.5 (OD_600_). The cell suspensions (100 μL) were mixed with 1.5 μL of PI (1 mg/mL) and incubated at 30°C for 5 min. Finally, cells were washed with PBS and resuspended in 100 μL of PBS. The fluorescence intensity was measured by a SYNERGYTM4 multifunctional enzyme mark instrument (Bioteck S.P.A., Italy) at the excitation and emission wavelengths of 535 and 617 nm, respectively. Each experiment was conducted in triplicate.

(1) $$\begin{array}{l} \text{Membrane disrupting activity }=\frac{\text{fluorescence intensity}}{\text{O}{\text{D}}_{600}} \end{array}$$

### BC harvest

Harvest of BC was described as before (Zhong et al., 2013). In brief, BC produced at anode and cathode sides was harvested, respectively. It was rinsed with distilled water and then placed immediately in NaOH (0.1 mol/L) to remove attached media and bacterial cells. Then BC was extensively washed in distilled water until the pH was neutral. And then the BC was dried at 80°C for 10 h until stable weight. The dry weight was recorded for each pellicle at room temperature.

### Quenching, extraction, and derivatization

The quenching and extracting processes were performed according to the methods of Villas-Bôas with some modifications (Villas-Bôas et al., 2005). Samples were immediately quenched by a prechilled (−40°C) methanol solution (60.0% v/v, methanol/water) to arrest metabolism instantaneously. Then they were centrifuged at 4000 rpm for 5 min to harvest cells. To remove the residual medium and salts, cells were washed with PBS and ultrapure water, respectively. Cell pellets were collected by centrifugation at 4000 rpm for 5 min, and were then grounded to a fine power in liquid nitrogen. Cell powder (50.0 mg) was suspended with 1 mL of methanol/water (1:1, v/v, −40°C) for metabolite extraction, and was thoroughly mixed by a vortex mixer. The mixture was frozen in liquid nitrogen and thawed for 4–5 times. The supernatant was obtained for metabolic analysis after centrifugation at 10,000 rpm for 5 min. To correct minor variations occurring during analysis, internal standard (1.4 mg/mL succinic acid, 2,2,3,3-*d*_**4**_, 10.0 μL, Sigma) was added to 300 μL of extracting solution before lyophilization. Four replicates were performed for each sample.

Derivatization of samples: firstly, the freeze-dried residue was redissolved and derivatized at 40°C for 80 min with 50 μL of methoxyamine hydrochloride (20.0 mg/mL in pyridine, Sigma) for methoximation of the carbonyl groups. Then a treatment at 40°C for 80 min with 80 μL of N-methyl-N-(trimethylsilyl) trifluoroacetamide (MSTFA, Sigma) was performed for trimethylsilylation. And then the mixture was centrifuged at 10,000 rpm for 5 min, the supernatant was used for GC-MS analysis.

### Detection of metabolites by GC-MS

The GC-MS system was consisted of an Agilent 7890A gas chromatography (GC) system and a 5795C quadrupole mass selective detector (Agilent Technologies, Palo Alto, CA). GC was performed on a HP-5 column (60 m × 0.32 mm i.d. × 0.25 μm, Agilent Technologies). A total of 1 μL of the derivatized sample was injected, and the split ratio was 1:10. The temperatures of injector, GC interface, and ion source were set at 280, 270, and 250°C, respectively. The oven temperature was initially held at 70°C for 2 min, increased to 290°C with a gradient of 5°C/min, and was then held at 290°C for another 3 min. Helium was the carrier gas with a constant flow of 1 mL/min. Mass spectra were recorded at two spectra per second with an m/z 50–800 scanning range (Liu et al., 2015).

Mass spectral peak identification and quantification were performed by MESCHEM software (Agilent Technologies). Peak deconvolution was performed by AMDIS-32 previously. The NIST MS standard reference databases 2.0 were used for the identification of metabolites.

### Data analysis

The relative content of detected metabolite was expressed as the ratio of each metabolite's GC peak area to that of the internal standard in the same chromatogram (2). Normalized peak areas were imported into SIMCA-P for the multivariate statistical analysis. Principal component analysis (PCA), partial least-square analysis (PLS), and hierarchical clustering analysis (HCA) were performed with the orthogonal signal correction (OSC) preprocessing.

(2) $$\begin{array}{l} \text{R}{\text{T}}_{\text{m}}=\frac{{\text{A}}_{\text{m}}}{{\text{A}}_{\text{i}}\times {\text{W}}_{\text{S}}\;} \end{array}$$

In which, RT_m_—relative content of metabolite; A_m_—GC peak area of metabolite;

A_i_—GC peak area of internal standard; and W_s_ is the weight of sample powder (50.0 mg).

## Results

### The 16S rDNA genes and phylogenetic analysis of *G. xylinus* CGMCC 2955

**Figure 1:**
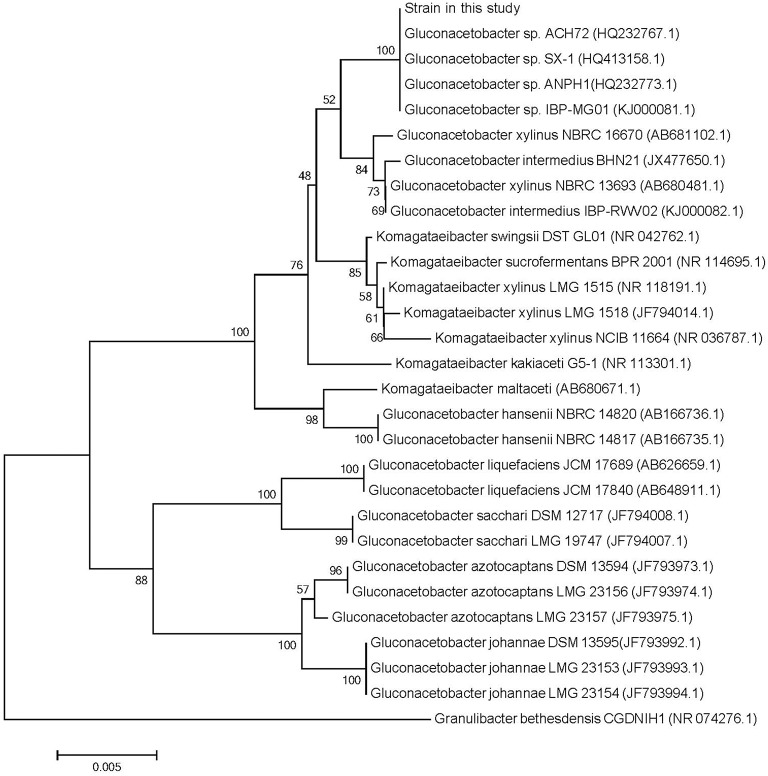
**A phylogenetic tree based on 16S rRNA gene sequences for Gluconacetobacter sp. and Komagataeibacter sp**. The phylogenetic tree derived from the neighbor-joining methode was newly constructed (Yamada et al., 2012). The type strain of *Granulibacter bethesdensis* (HR 074276.1) was used as an outgroup. The numberals at the respective branching points indicate bootstrap values (%) based on 1000 replications.

The phylogenetic tree obtained with the 16S rDNA sequence data (Supplementary Data Sheet 2) of the *Gluconacetobacter* sp. and *Komagataeibacter* sp. was illustrated in Figure 1. The *G. xylinus* and *Gluconacetobacter intermedius* strains clearly constituted a cluster separate from the clusters formed by the strains of *Komagataeibacter xylinus, Komagataeibacter swingsii*, and *Komagataeibacter sucrofermentans*. Phylogenetic trees of the strain in this study and reference strains of the *Gluconacetobacter* sp. were rather similar to each other. This analysis confirmed that strain in this study was a member of the *G. xylinus* species group.

### The growth of *G. xylinus* CGMCC 2955 and BC production

**Figure 2:**
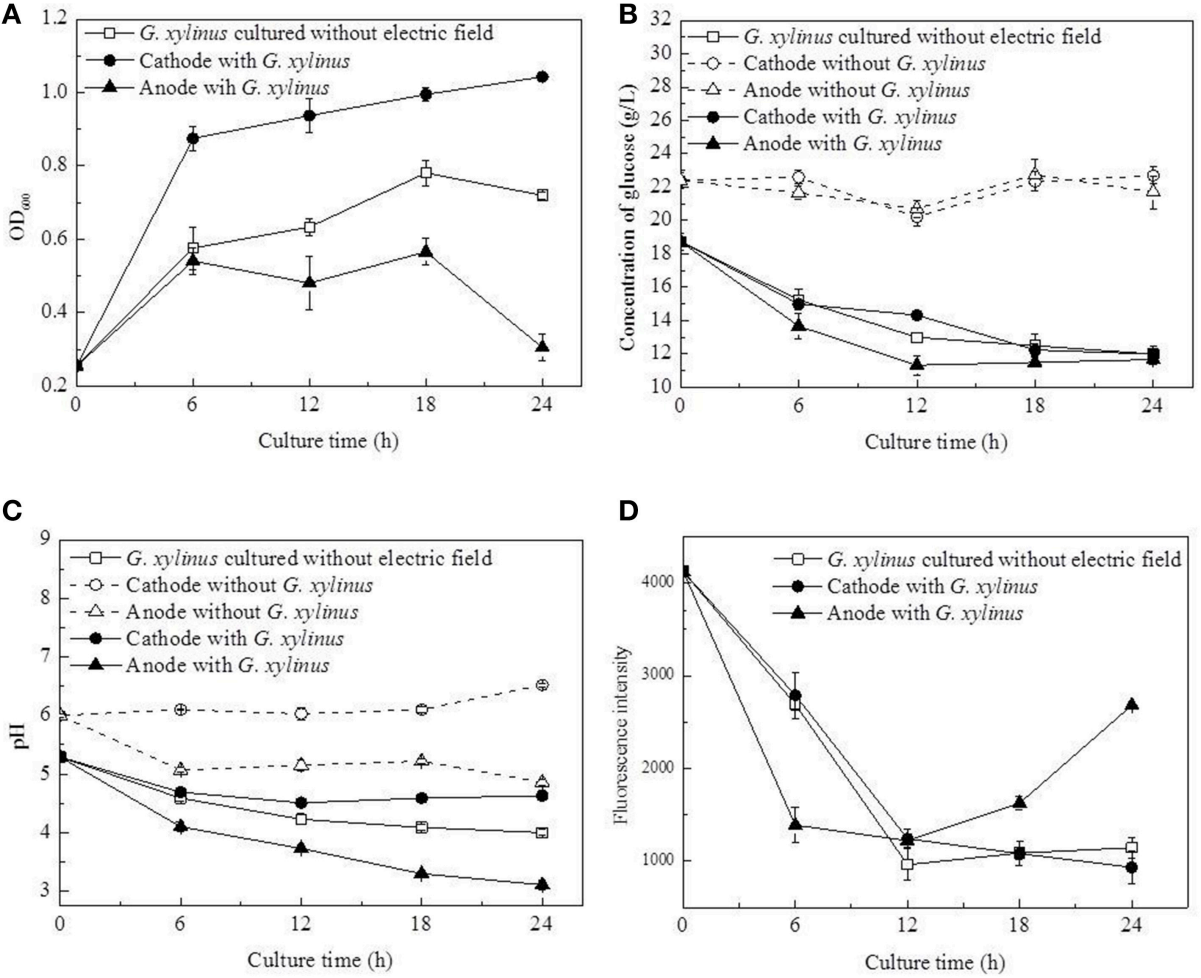
**Changes of cell density, pH, glucose concentration, and membrane-disrupting activity of ***G. xylinus*** cultured with/without DC electric field**. (**A**, Cell density; **B**, Concentration of glucose; **C**, pH; **D**, Membrane disrupting activity). Dash lines in **(B,C)**: electrolysis testing in the fresh medium; Data represent means ± SD from four independent experiments.

The growth curves of *G. xylinus* were observed to be different between the cathode and anode as shown in Figure 2A. At the cathode, the cell density increased to 1.52-fold at 6 h compared to the control (without DC electric field). In the next 18 h, cell density was increased at a lower rate from 0.88 to 1.04. At the anode, the cell density was not greatly affected in the first 6 h of DC treatment as compared with the control. However, in the next 18 h, cell density remained unchanged before declining at 18 h to 53.89%. It was likely that, at the anode, cell growth was inhibited since 6 h. Glucose consumption (Figure 2B) also indicated that cells at the anode barely consumed carbon source since 12 h.

Compared with the control group, the pH value showed a distinct changing pattern in DC treated group, in which it decreased from 5.29 to 4.51 in 12 h followed by a slightly increase to 4.63 in 12–24 h at the cathode (Figure 2C). Besides, it has to been noticed that the pH at the anode decreased to 3.3 in 18 h, which was beyond the optimal pH range (4.0–6.0) of *G. xylinus* CGMCC 2955. When the initial pH dropped from 4.0 to 3.0, the BC yield could be decreased by more than 76.92% (data not shown). So it might be one of reasons for cell growth inhibition or even death at the anode. To determine how the pH value was affected by water electrolysis with growth medium in DC electric field, DC was applied to fresh culture broth without any *G. xylinus* inoculated. As shown in Figure 2C, hydrogen ion (H^+^) produced at the anode decreased the pH of culture broth to 4.86 at 24 h. While hydroxide ions (OH^−^) produced around the cathode could neutralize the organic acid produced by *G. xylinus*, resulting in a slightly pH change throughout the culture process. This stable acid-environment was beneficial for *G. xylinus* growth (Figure 2A).

Concentrations of glucose in fresh medium under DC electric field were seen in Figure 2B. No significant changes were observed as culture time progressed, confirming that glucose was stable in the presence of the DC electrode reactions.

The membrane-permeabilizing activity of *G. xylinus* was investigated using the PI assay (Figure 2D). In the control group and DC treated groups at the cathode, the fluorescence intensities were approximately equal, and they all decreased significantly as the culture time extended. At the anode, fluorescence intensity was 49.42% lower than that of the control group at 6 h. However, it reached to 2.34-fold of that of the control group at the end of DC treatment.

**Figure 3:**
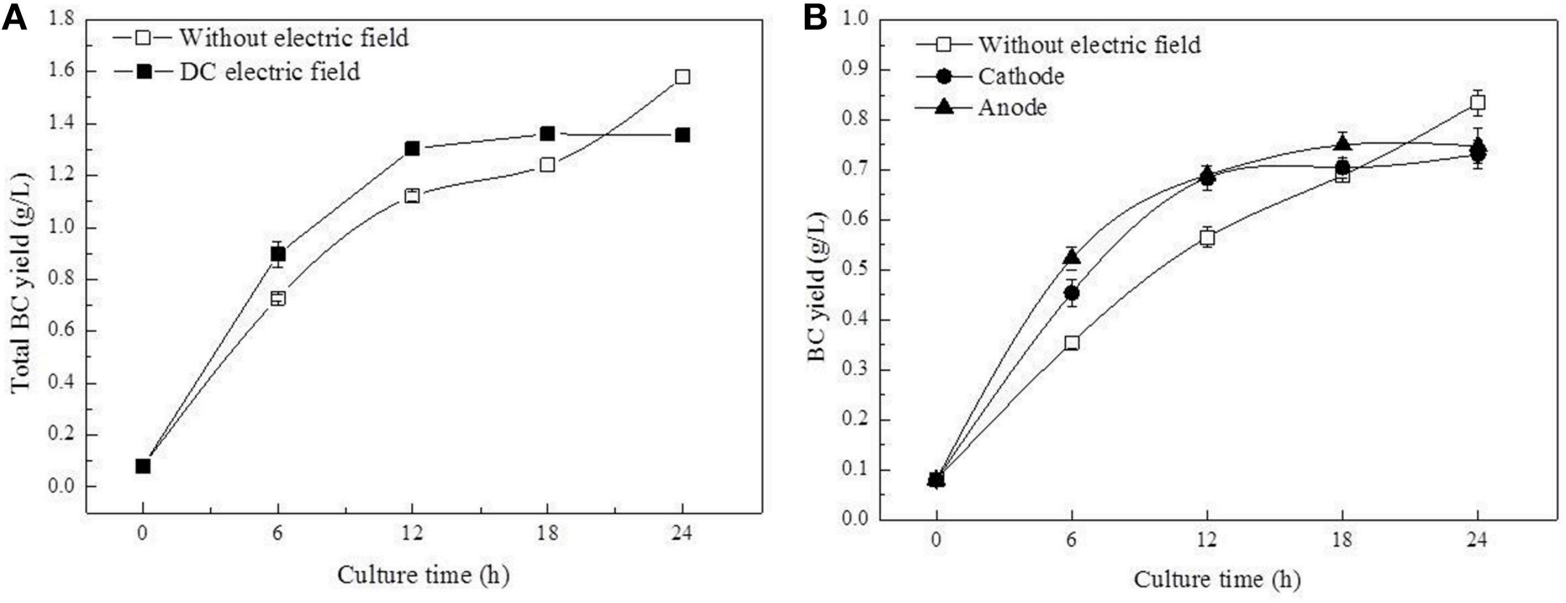
**BC yield of ***G. xylinus*** cultured with/without DC electric field**. (**A**, Total yield of anode and cathode; **B**, Anode/Cathode yield).

With DC treatment, the total BC yield (BC yield of cathode and anode) increased from 0.08 to 1.30 g/L in 12 h before it leveled off at 24 h (Figure 3A). Further analysis revealed that BC yield at the anode was relatively higher than that at the cathode (Figure 3B). In the control group, less BC was obtained compared to the DC-treated group at 18 h (the total BC yield), but the highest BC yield was obtained at 1.58 g/L at the end of culture process.

### Effect of DC electric field on intra-cellular metabolic profiles of *G. xylinus*

A total of 104 compounds were identified in both the control and DC treated groups. These metabolites mainly included amino acids and derivatives, sugars, alcohols, organic acids, amines, and unknown compounds.

**Figure 4:**
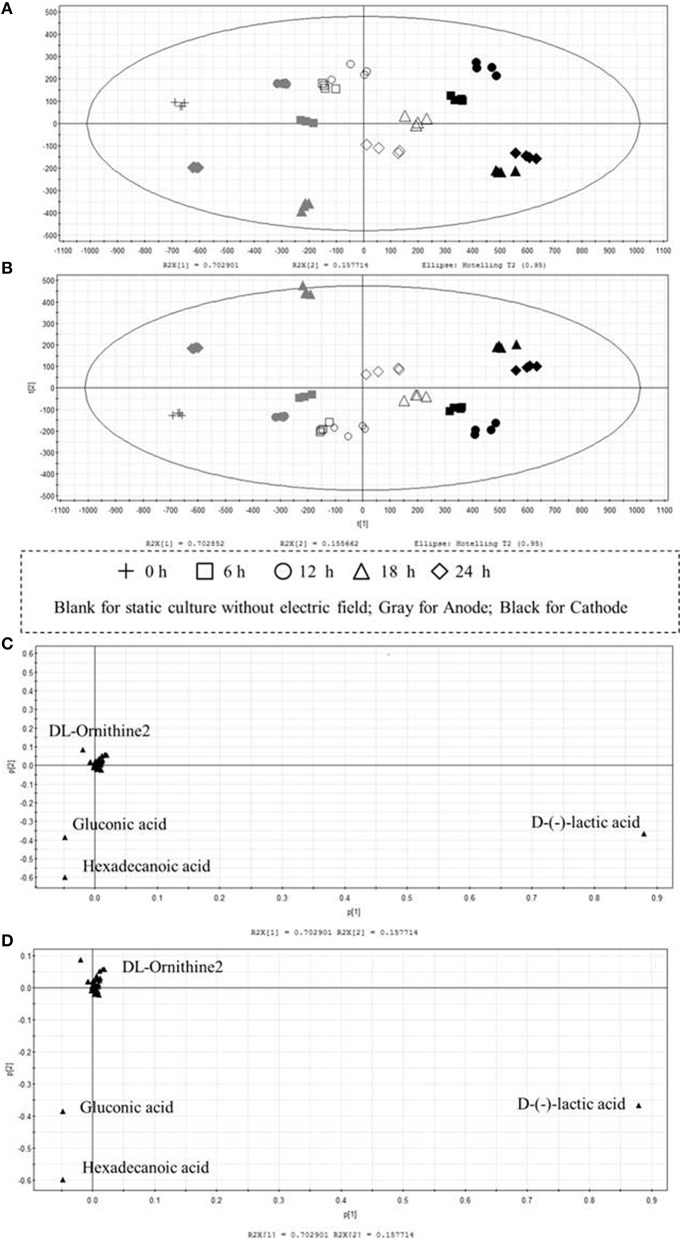
**Multivariate statistical analysis of intra-cellular metabolites of ***G. xylinus*** cultured with/without DC electric field**. (Score plot **A**, PCA; **B**, PLS; Loading plot **C**, PCA; **D**, PLS). In the scores plot, the confidence interval is defined by the Hotelling's T2 ellipse (95% confidence interval), and observations outside the confidence ellipse are considered outliers.

Multivariate data analysis was performed by PCA and PLS to examine the variations of intra-cellular metabolites in cells cultured with/without DC electric field. Both models were well constructed with excellent fit and satisfactory predictive capability. PCA, an unsupervised clustering method, was mainly used for exploring an overview of the GC-MS data and investigating the metabolic differences between DC treated cells and the control cells. Samples cultured with/without DC electric field were separated clearly by PCA as shown in Figure 4A. The first principal component (PC1) that accounted for 70.29% of the total variations among the experimental groups clearly separated the samples into three groups: samples from the anode, control groups, and samples from the cathode. Compared with the control, metabolic profiles of *G. xylinus* from the anode were more similar with that at 0 h (before DC electric field was introduced). This was consistent with what was illustrated in Figure 2A. Meanwhile, the HCA plot of the 104 metabolites reflected a clustering pattern that was similar to the result of PCA (Figure S3). PLS analysis, a supervised clustering method, was performed to further validate the differences among the control, and DC treated groups. As illustrated in Figure 4B, PLS also supported a clear discrimination among all the groups, getting a similar clustering to that of the PCA and HCA. It indicated that DC electric field dramatically changed the intra-cellular metabolism of *G. xylinus*.

To identify the metabolites mainly responsible for discriminating among cells cultured with/without DC electric field, the contribution of each metabolite to metabolic differences was analyzed by loading plots in PCA and PLS (Figures 4C,D). In the PLS models, the VIP coefficient reflects the contribution of each metabolite. The higher VIP value means that the metabolite has a larger contribution. A metabolite with a VIP value >1 suggests a major contribution to discriminating groups within PLS models. D-lactic acid, hexadecanoic acid, and gluconic acid were found to be the most influential metabolites that were responsible for the distinction between DC treated cells and the control cells by both PCA and PLS.

To evaluate the significant differences of metabolites between DC treated groups and that in the control group, a total of 30 identified typical metabolites were analyzed (Table 1). These metabolites were divided into different categories based on the type of compounds and their positions in the metabolic pathway, such as central carbon metabolism, amino acid, membrane structure, organic acid, and alcohols.

**Table 1:** **The intra-cellular metabolites of ***G. xylinus*** CGMCC 2955 cultured with/without DC electric field**.

	**Retention time**	**Static culture without electric field**					**Cathode**				**Anode**			
		**0 h**	**6 h**	**12 h**	**18 h**	**24 h**	**6 h**	**12 h**	**18 h**	**24 h**	**6 h**	**12 h**	**18 h**	**24 h**
D-citramalic acid	25.780	10.64	21.53	30.13	47.57	23.03	20.20	16.23	24.83	22.34	13.97	10.53	13.99	8.29
d-erythrotetrofuranose	32.532	8.32	277.96	51.80	86.95	0.00	336.94	42.46	23.61	28.89	27.72	10.23	9.39	10.97
d-glucose	34.403	118.38	124.70	120.45	71.32	65.97	94.21	89.35	73.86	32.23	47.39	47.30	100.91	47.25
d-gluconic acid	34.073	36.90	28.94	281.16	161.03	287.49	37.26	43.09	312.66	129.85	34.92	145.03	1165.78	700.69
D-(-)-lactic acid	12.293	435.20	2682.62	4546.49	4727.81	3495.36	2287.65	2691.10	4521.89	5645.26	1706.15	1021.63	2307.36	1318.29
DL-orinithine	30.380	53.34	117.55	28.24	10.72	10.78	13.07	17.51	17.72	11.28	9.96	9.80	10.03	3.50
DL-ornithine2	31.844	124.01	601.13	120.69	37.77	24.50	115.48	40.51	40.41	14.77	57.90	15.44	35.46	12.36
D-mannitol	36.009	ND	ND	30.93	34.70	42.60	21.90	41.41	24.82	23.64	0.00	12.49	30.03	18.52
Ethyl alcohol	12.681	4.10	11.18	18.19	41.53	16.38	12.02	14.89	21.67	20.70	15.15	8.00	9.69	6.42
Glyceric acid	19.963	20.37	69.39	98.16	92.93	69.20	91.80	139.88	70.05	53.51	63.85	111.66	50.33	35.95
Glycyl-l-glutamic acid	25.021	41.02	76.68	114.01	152.89	75.76	119.07	117.84	101.88	64.53	47.53	108.74	105.37	72.47
Gulonic acid	34.113	ND	ND	ND	ND	ND	ND	ND	28.83	23.06	ND	19.76	92.54	43.13
Hexadecanoic acid	37.385	163.50	164.58	839.75	589.10	653.70	285.30	334.84	962.66	610.48	392.94	592.53	2163.87	1678.82
L-aspartic acid 2	21.651	24.34	25.07	60.48	83.06	59.93	44.71	60.85	45.86	22.06	12.38	56.20	64.09	33.01
L-citrulline	30.568	6.01	377.71	14.55	52.89	5.24	476.03	114.67	15.93	11.05	57.33	6.17	93.54	154.66
L-proline 1	24.886	73.34	218.94	407.30	323.59	168.84	174.95	142.74	168.36	103.68	139.33	202.21	226.39	123.79
L-threonine	18.910	38.64	75.39	81.71	118.64	48.06	96.82	93.14	68.66	74.01	34.95	66.75	39.68	24.78
L-valine	12.998	5.74	24.56	23.65	43.16	14.73	15.62	11.10	18.34	14.75	10.71	9.53	12.17	5.58
N-α-acetyle-l-lysine	32.685	7.26	155.81	42.82	46.03	18.22	70.10	47.13	29.27	29.53	35.64	21.92	14.05	7.34
n-octanoic acid	26.350	2.61	6.93	8.88	19.75	4.78	17.37	21.59	10.19	18.82	2.84	11.40	3.48	3.89
N,O,O-aspartic acid	23.709	6.19	10.17	6.68	25.51	9.95	19.42	13.47	9.13	13.50	3.43	7.67	3.60	2.72
Octadecanoic acid	43.726	84.66	143.10	227.88	228.69	209.94	163.84	154.71	170.48	173.35	247.38	165.17	147.36	202.71
Phosphate	18.410	1135.56	3043.65	4984.39	3852.51	2744.01	3460.85	4225.39	3243.56	3692.35	3530.00	3000.63	2725.00	2124.54
Phosphoric acid	15.681	13.91	116.21	180.30	128.57	105.44	80.43	123.14	55.31	85.10	35.31	99.67	84.41	89.29
Pyrimidine	20.133	12.28	24.45	43.48	45.23	29.34	34.16	41.00	26.35	15.10	30.62	21.20	13.50	9.92
Tetradecanoic acid	32.279	25.78	32.24	ND	4.32	44.97	128.78	23.83	23.27	30.44	63.17	27.83	20.06	28.22
1,2-ethanediol	10.175	103.38	103.38	103.38	103.38	103.38	103.38	103.38	103.38	103.38	103.38	103.38	103.38	103.38
2-pentanoic acid	37.128	9.97	9.55	10.45	8.26	6.64	10.83	14.31	8.05	14.34	ND	ND	ND	ND
3-phospho-glycerol	30.974	35.74	352.59	154.29	151.22	110.20	342.94	146.66	96.84	75.49	191.37	60.12	103.00	59.73
9H-purin-6-amine	33.097	7.55	38.41	47.59	34.60	10.83	52.99	21.98	14.02	10.08	0.00	2.87	6.50	3.28

### Variations of intra-cellular metabolites

**Figure 5:**
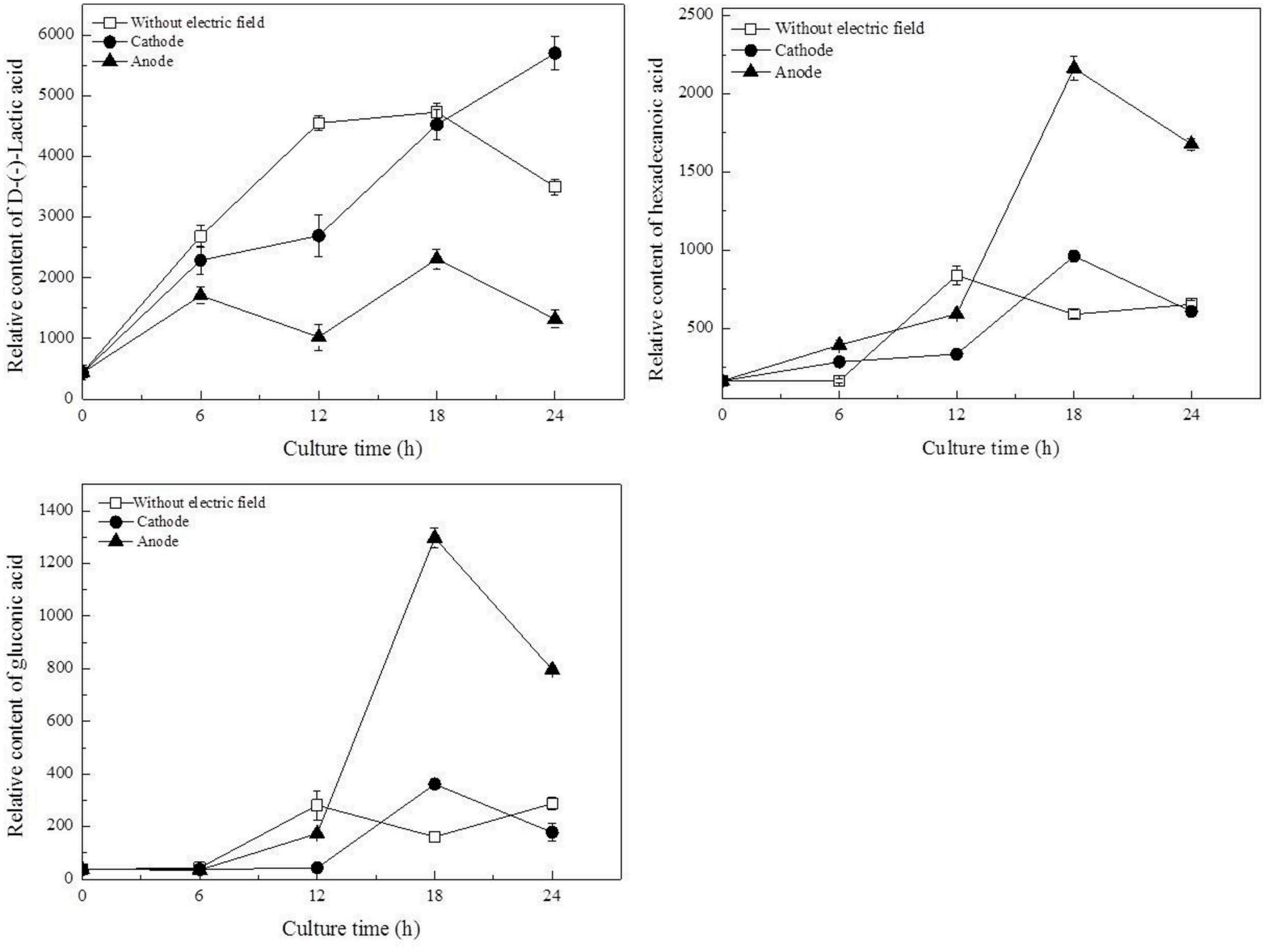
**Potential biomarkers for discrimination of samples from the control and DC treated groups**. Data represent means ± SD from four independent experiments.

As illustrated in Table 1, glucose, as the origin of glycolysis, was found to decline in DC treated cells, especially those at the anode in the first 12 h of DC treatment. Then at 18 h, where cell density turned to decrease at the anode, glucose was accumulated to 2.13-fold compared with that at 12 h. And then it decreased to 46.81% at 24 h. Gluconic acid was a potential biomarker to distinguish the control samples from the DC treated ones. It is one of sugar-derived C6-acids and is also the main by-product of *G. xylinus* from glucose, which is mainly involved in pentose phosphate pathway during BC production. As shown in Figure 5, gluconic acid at the anode was found to be 7.97-fold of that in the control at 18 h. Then it sharply decreased by 38.64% at 24 h. The α-ketoglutaric acid and butanedioic acid are related to the TCA cycle. At the anode, the contents of intra-cellular α-ketoglutaric acid and butanedioic acid decreased dramatically to 49.23 and 56.36% at 6 h as compared with the control (**Figure 8**). Both of them increased until 18 h, and then decreased sharply at 24 h. On the contrary, as compared with the control and the anode groups, the levels of butanedioic acid in cells from the cathode increased to 1.80- and 3.42-fold at 12 h. And α-ketoglutaric acid was accumulated at 18 h with a similar level to the control.

Of all the detected organic acids, D-lactic acid was the most notable potential biomarker contributing to discrimination (Figures 4C,D). The relative contents of D-lactic acid were shown in Figure 5. Compared with the control, cells at the cathode displayed an excessive accumulation of D-lactic acid to 1.63-fold at 24 h. In contrast, D-lactic acid was relatively lower in *G. xylinus* at the anode. But the highest content was also obtained at 18 h.

**Figure 6:**
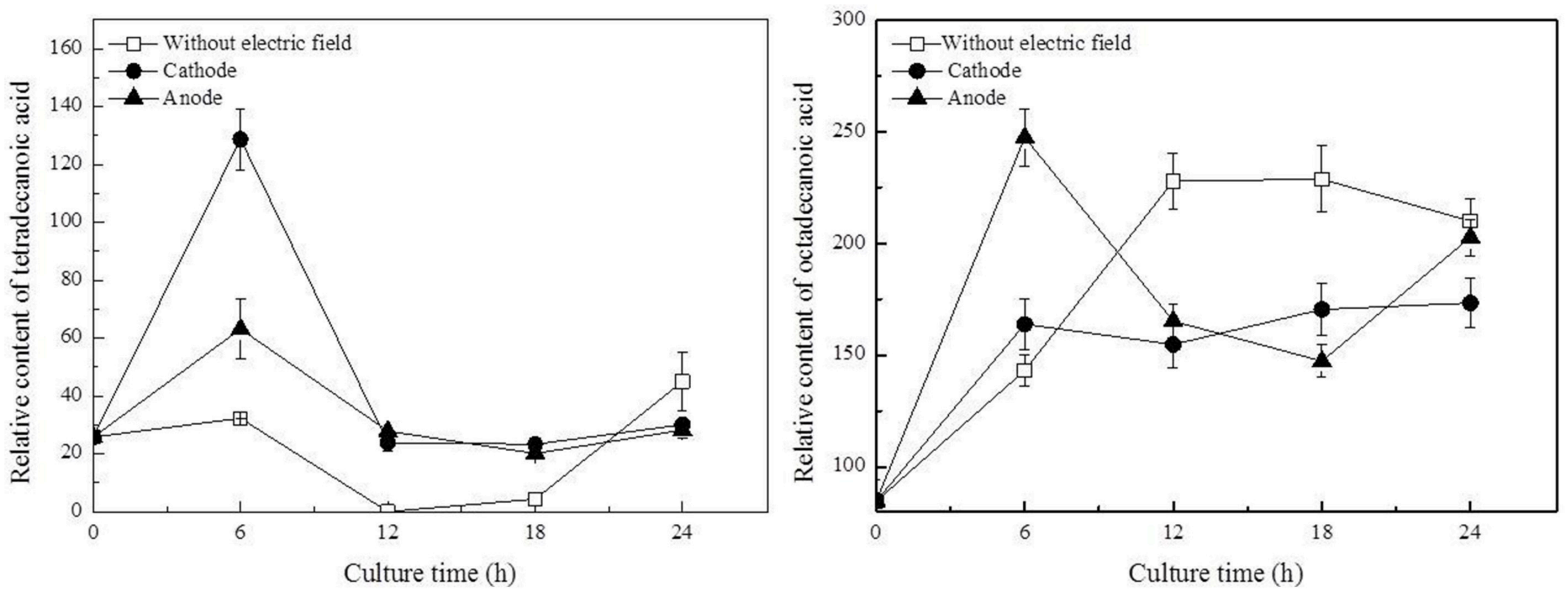
**Variations of fatty acids in ***G. xylinus*** under DC electric field treatment**. Data represent means ± SD from four independent experiments.

**Figure 7:**
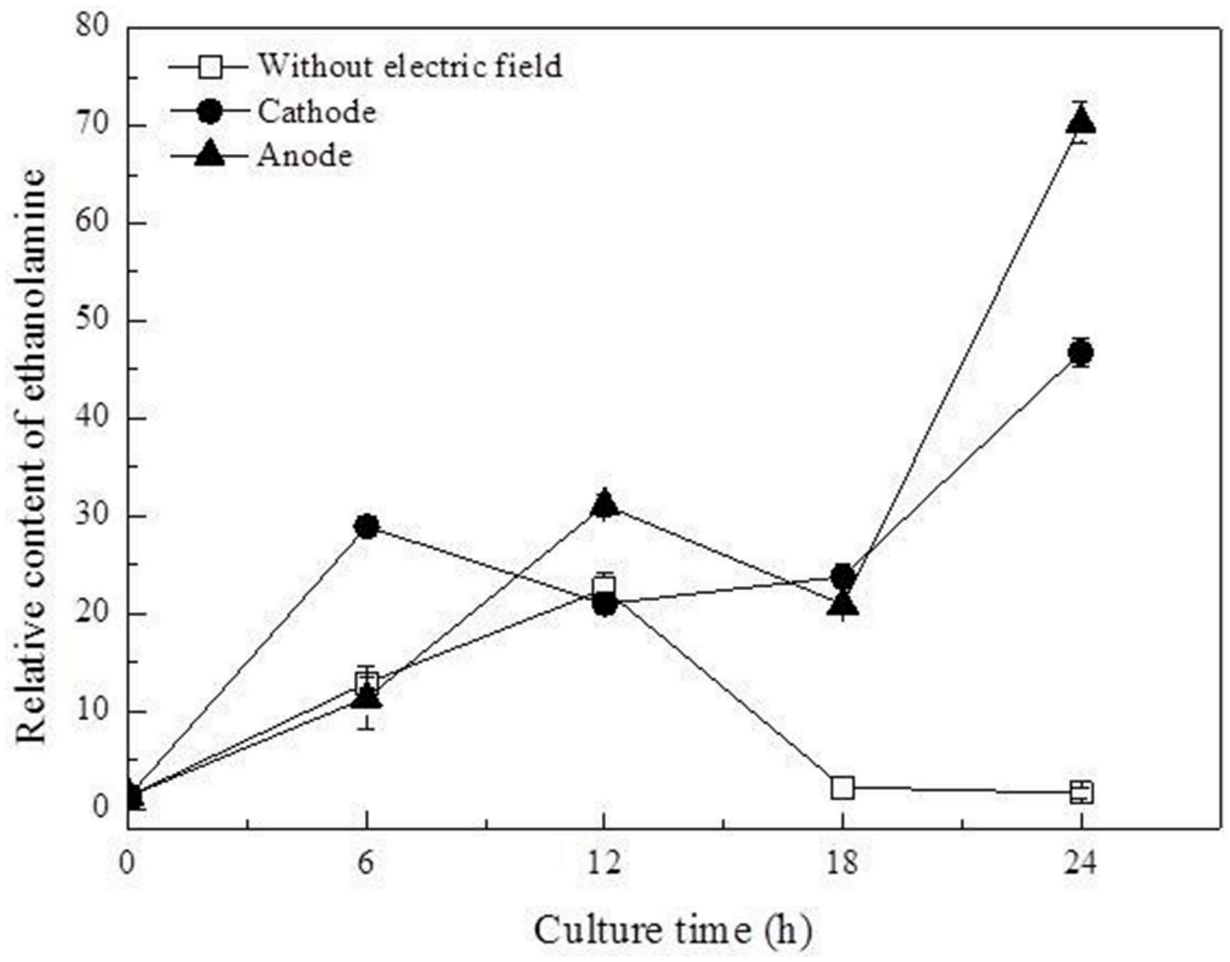
**Variation of ethanolamine contents in ***G. xylinus*** cultured with/without direct current electric field**.

In this work, three saturated fatty acids were detected, including tetradecanoic acid, hexadecanoic acid (Figure 5), and octadecanoic acid (Figure 6). Hexadecanoic acid was found to be one of the biomarkers for discrimination of *G. xylinus* samples cultured either with or without DC electric field. *G. xylinus* in the control group were much inferior to those with DC treatment in the relative contents of tetradecanoic acid and octadecanoic acid in the first 12 h. Furthermore, hexadecanoic acid showed the exactly same changing patterns with gluconic acid. Compared with the control, hexadecanoic acid level in cells at the anode was significantly rose by 2.67-fold at 18 h. Fatty acids are closely related to membrane fluidity, and ethanolamine is the precursor of phospholipid biosynthesis (Dupont et al., 2011). As shown in Figure 7, intra-cellular ethanolamine contents in DC treated groups increased with culture time.

## Discussion

The morphology, movement and BC productivity of *G. xylinus* in electric field were studied in our previous work (Zheng et al., 2014). In that case, with 0.5 V/cm (10 mA), a distinct BC yield, and cell density were obtained between the anode and cathode. Besides, another study (She et al., 2006) indicated that the best stimulating effects in terms of cell growth and the dehydrogenase activity were when a DC of 10 mA was applied for 12 h. Diao (Diao et al., 2004) also pointed out that the current density should be kept under 20 mA to prevent bactericidal action. In this work, the current density was kept at 10 mA.

**Figure 8:**
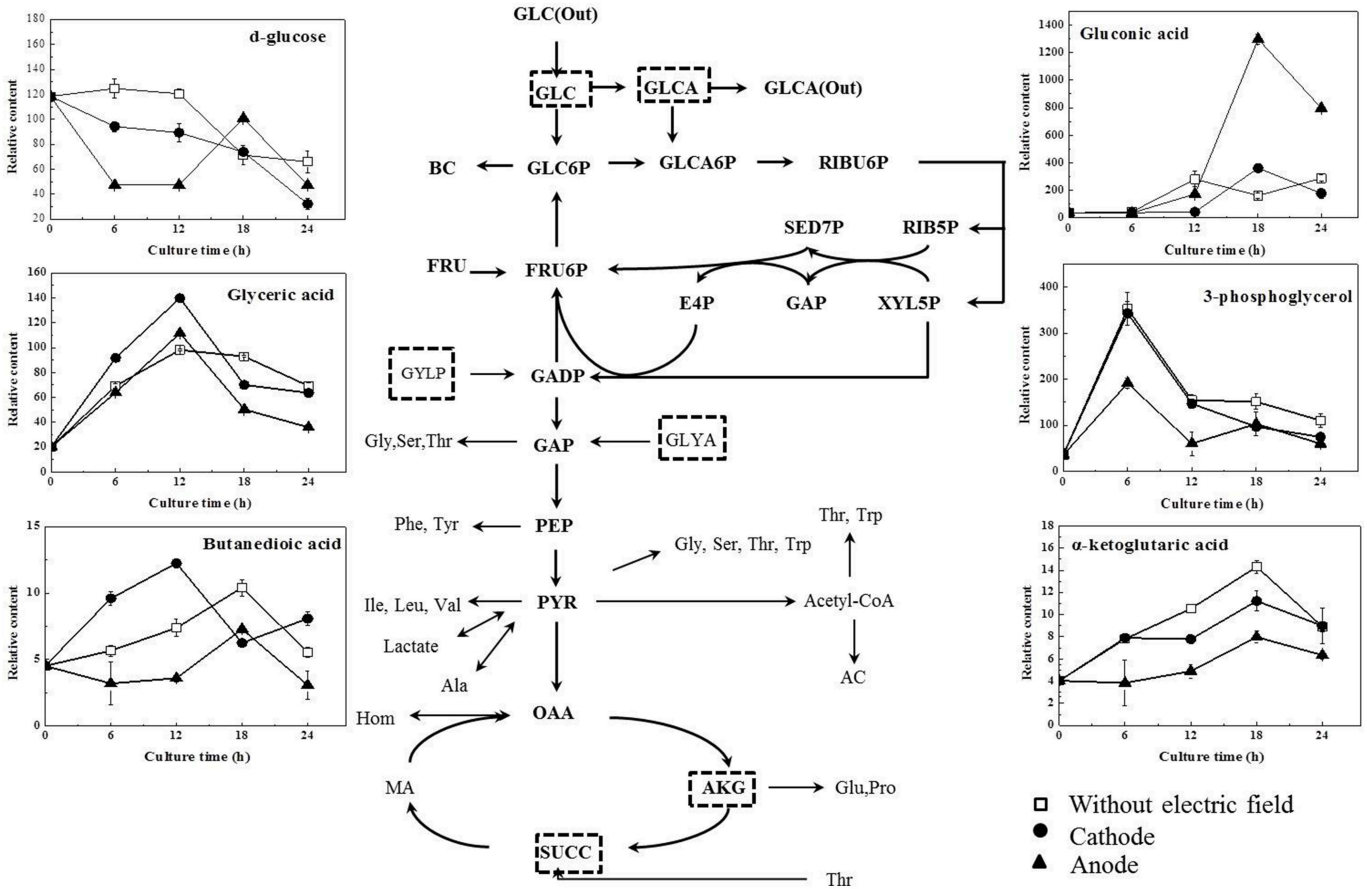
**Overall schemes of metabolites relative content changes mapped onto the metabolic network**. (Diamonds, without electric field; round, cathode of DC electric field; triangle, anode of DC electric field). GLC, glucose; GLCA, Gluconic acid; BC, Bacterial Cellulose; UDPG, Uridine diphosphoglucose; GLC6P, Glucose-6-phosphate; RIBU5P, Ribulose-5-phosphate; RIB5P, Ribose-5-phosphate; XYL5P, Xylulose-5-phosphate; SED7P, Sedoheptulose-7-phosphate; E4P, Erythrose-4-phosphate; FRU6P, Fructose-6-phosphate; G3P, 3-Phosphoglycerate; GAP, Glyceraldehyde-3-phosphate; G3P, glycerol-3-phosphate; GLYA, glyceric acid; PEP, Phosphoenol pyruvate; PYR, Pyruvate; AC, Acetate; Ser, serine; Leu, leucine; Val, valine; Hom, homoserine; Glu, glutamic acid; Pro, proline; FA, fatty acid; HEX, hexadecanoic acid, OCT, octadecanoic acid; OCTE, octadecenoic acid; ACCOA, Acetyl-coenzyme A; OAA, Oxaloacetate; AKG, α-keto-gluterate; SUCC, Succinate.

In this work, DC electric field significantly affected BC production and cell metabolism as seen in Figures 2, 4. Previous reports indicated that the activation or inhibition of specific function can be reflected by amounts of related metabolites (Jozefczuk et al., 2010; Bo et al., 2014; Liu et al., 2015). To evaluate the dynamic changes of metabolic networks (Zhong et al., 2013, 2014) in *G. xylinus*, the measured intra-metabolites were mapped onto the *G. xylinus* biosynthetic pathways (Figure 8). At the cathode, the significant decrease of intra-cellular glucose contents and increase of glyceric acid and butanedioic acid contents in the first 12 h indicated that both the glycolysis and TCA cycle pathways were activated compared to the control (Figure 8). On the contrary, at the anode, the activities of both the glycolysis pathway, and the TCA cycle were inhibited during 18–24 h (Figure 8). These results were consistent with cell density and PI assay results. Metabolic analysis revealed that some metabolites were greatly accumulated at 18 h, including proline, glutamic acid, hexadecanoic acid, gluconic acid, and lactic acid.

Proline is synthesized from glutamic acid, and it can also be converted to glutamic acid. They are both important amino acids in terms of their biological functions in response to various stress conditions (Lu et al., 2013). Our previous work also revealed their functions in *G. xylinus* as cell protectors against hydrodynamic stresses (Liu et al., 2015). In this work, glutamic acid and proline were both significantly accumulated at 18 h (Figure S4). Meanwhile, as shown in Figures 5, 8, the levels of gluconic acid increased upon exposure to DC at the anode. It was also reported to be stress protection (Gheldof et al., 2002; Bo et al., 2014).

PI assay confirmed that membrane permeability of cells at the cathode was increased during 18–24 h. Dupont et al. indicated that the dynamic of membrane components, reflecting in changes of membrane fluidity could be affected by environmental condition (Dupont et al., 2011). The unsaturated to saturated fatty acids (UFA/SFA) ratio was used as an indirect indicator of the membrane fluidity (Casadei et al., 2002). Increase in SFAs levels to some degree indicated the decrease of membrane fluidity of cells exposed to DC (Figure 6; Álvarez-Ordóñez et al., 2008). Ethanolamine, as a precursor of phospholipid biosynthesis, was found to increase as culture time extended in DC treated groups (Figure 7). It also indicated that DC induced the changes in membrane fluidity of *G. xylinus*. And this might be a response to the changes of environmental conditions, such as in anodic intermediates (She et al., 2006), temperature (Casadei et al., 2002), or pH (Yuk and Schneider, 2006).

The cyclic voltammetry of DC electric field with the Pt electrodes indicated that water electrolysis was the major electrode reaction (She et al., 2006). Hydrogen and oxygen were the two major products.

$$\begin{array}{l} \text{Anode:}2{\text{H}}_{2}\text{O}\to {\text{O}}_{2}+4{\text{H}}^{+}+4\text{e}; \end{array}$$

$$\begin{array}{l} \text{Cathode:}4{\text{H}}_{2}\text{O}+4\text{e}\to 2{\text{H}}_{2}+4\text{O}{\text{H}}^{-}; \end{array}$$

It is well known that appropriate oxygen supply can enhance the BC production in *G. xylinus* (Liu et al., 2015). However, in this work, the increased fluorescence intensity indicated that the anode led to an accelerated cell death during 18–24 h of DC treatment (Figure 2D). Besides, both cell growth and BC production were found to be increased at the cathode compared to the anode and the control group (as seen in Figures 2A, 3). Our results were contrary with that of Sano (Sano et al., 2010), which indicated that there might be some other factors mainly affecting *G. xylinus* growth instead of oxygen. As for the anode, it was possibly due to the presence of anodic intermediates including H^+^, H_2_O_2_, and O${}_{2}^{-}$ (She et al., 2006), or the pH of electrolytic biomass fluid that could have been the contributing factor for the bacterial inactivation (Choi et al., 2014). With regard to the cathode, it was claimed that the cell growth promotion was likely to be related to the generated hydrogen, which functions as a proton donor to stimulate the dehydrogenase system (She et al., 2006). Increasing hydrogen induced the reduction of the NAD/NADH, indicating a shift in the intracellular redox equilibrium toward the reduced state. This result is beneficial for the glucose utilization (Barron et al., 1997), which is consistent with the lower intra-cellular glucose contents and higher activity of glycolysis in cells at the cathode as compared to the control (Figure 8).

As for the anode, the temperature of the culture medium was observed to be only an increase of 0.2°C under the DC electric field. Therefore, the most possible factor that induced the response in cells at the anode might be a fluctuation of pH, which was found to be significantly decreased and far beyond the optimal pH range at the anode since 12 h (Figure 2C). Gluconic acid is the main by-product of *G. xylinus* when glucose is used as carbon source. The excessive accumulation of gluconic acid is not beneficial for BC synthesis since it costs lots of carbon source (Liu et al., 2015). The low cell viability and high gluconic acid levels were likely to be the main reasons for low BC production under a DC electric field during 18–24 h. The accumulated gluconic acid and other organic acids would notably drop the pH, which then resulted in cell viability decrease, metabolism suppression, and BC synthesis inhibition (Kashket, 1987; Seto et al., 1997). One of the organic acids—lactic acid was barely reported in *G. xylinus* that affected either cell growth or BC production. However, in this work, it was the most significant metabolite that contributed to discrimination between the control and DC treated groups (Figures 4C,D). Lactic acid is converted from pyruvic acid, a very important intermediate of glycolysis. Ding et al. reported that the pyruvic acid was partly transformed into lactic acid to generate energy (ATP) when there was not enough oxygen in *Saccharomyces cerevisiae* (Ding et al., 2009). This might explain the relatively high level of lactic acid in the control and the cathode samples, where dissolved oxygen was relatively lower than the anode. At 18 h, relative content of lactic acid at the anode was 2307.36, only 48.40% of that in the control and 51.03% of that at the cathode. Besides, it has to be noted that the extra-cellular pH was extremely low at the anode. It was reported that as a weak organic acid, lactic acid in its uncharged, protonated forms could diffuse across the cell membrane and dissociate inside the cell, and then decrease the internal pH (Ph_i_). The lower the external pH (pH_o_), the more undissociated latic acid would be available to cross the membrane and affected pH_i_. This meant that it took less organic acid to kill a cell at lower pH_o_ (Bearson et al., 1997). In other words, as the lactic acid was accumulated in the cytoplasm, the bacteria cells would suffer from an acid stress more tough than hydrochloric acid (Kashket, 1987). Sanders (Sanders et al., 1999) also claimed that intra-cellular pH decreased as extra-cellular pH was reduced, inducing the sharply decline of cell viability. Thus, it might be one of the reasons that the anode where less intra-cellular lactic acid was produced contributed to much higher cell mortality rate than the cathode. Therefore, it might be the extremely low extra-cellular pH (pH = 3.3 at 18 h) and excessive accumulated intra-cellular gluconic acid and lactic acid (Figures 5, 8) that made it a really tough acid stress at the anode, finally inducing a depression of *G. xylinus* growth in the last 6 h.

## Author contributions

ML carried out experiments, analyzed experimental results, and wrote this manuscript. CZ is the corresponding author, and he designed experiments and wrote this manuscript. YZ carried out some experiments. ZX carried out some experiments. CQ analyzed sequencing data and developed analysis tools. SJ is the corresponding author, and he designed experiments.

### Conflict of interest statement

The authors declare that the research was conducted in the absence of any commercial or financial relationships that could be construed as a potential conflict of interest.
