# Supplementary figures and images for: Metabolic Investigation in Gluconacetobacter xylinus and Its Bacterial Cellulose Production under a Direct Current Electric Field
Source: Front Microbiol. 2016 Mar 17;7:331. doi: 10.3389/fmicb.2016.00331 (PMC4794480; doi:10.3389/fmicb.2016.00331)

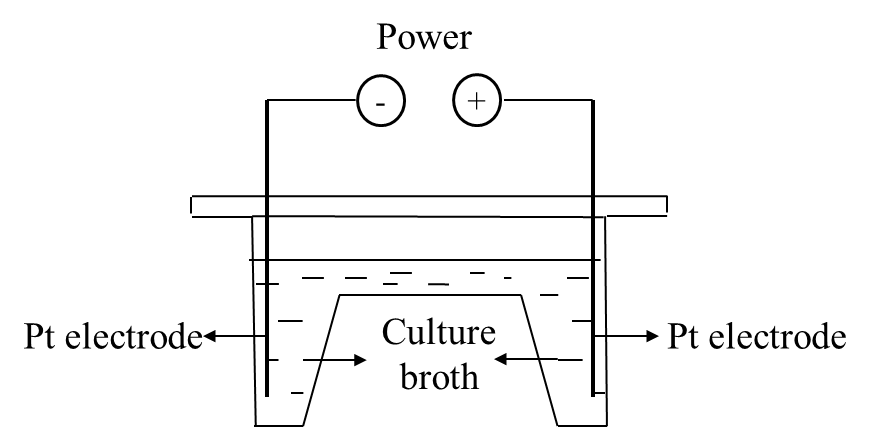


Fig. S1 Schematic figure of bioelectrical reactor system

Supplement: Supplementary file 1 [file DataSheet1.DOCX]

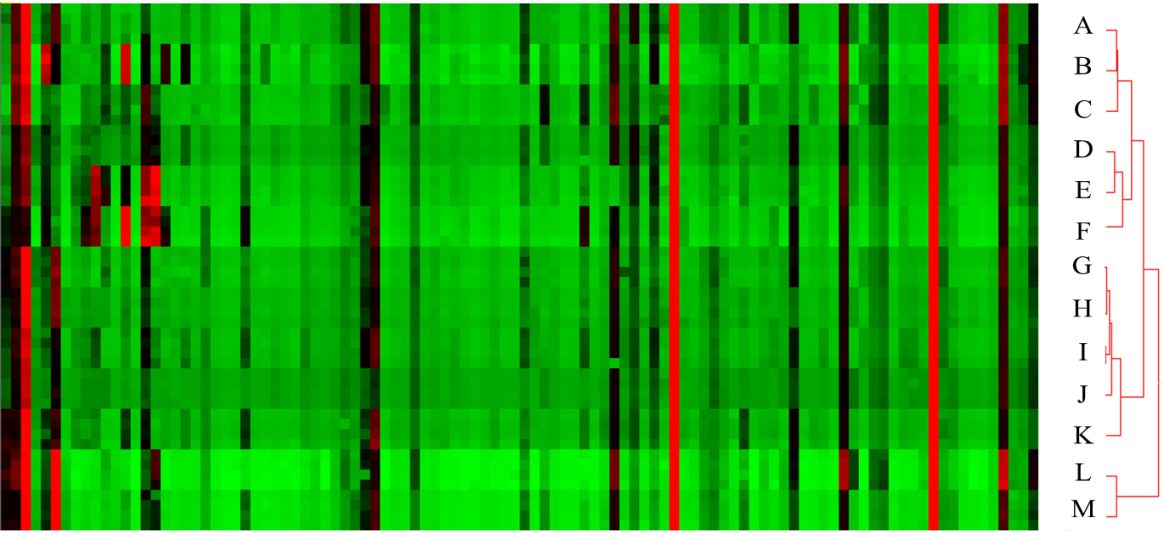


Fig. S3 Heat map of metabolites in G*. xylinus* cultured with/without DC electric field

Supplement: Supplementary file 3 [file DataSheet3.DOCX]
