# Supplementary material for: Metabolic Investigation in Gluconacetobacter xylinus and Its Bacterial Cellulose Production under a Direct Current Electric Field
Source: Front Microbiol. 2016 Mar 17;7:331. doi: 10.3389/fmicb.2016.00331 (PMC4794480; doi:10.3389/fmicb.2016.00331)
Supplement: Supplementary file 2 [file DataSheet2.DOCX]

16s rRNA sequence of *G. xylinus* CGMCC 2955

GCACGAACCTTTCGGGGTTAGTGGCGGACGGGTGAGTAACGCGTAGGGATCTGTCCATGGGTGGGGGATAACTTTGGGAAACTGAAGCTAATACCGCATGACACCTGAGGGTCAAAGGCGCAAGTCGCCTGTGGAGGAACCTGCGTTCGATTAGCTAGTTGGTGGGGTAAAGGCCTACCAAGGCGATGATCGATAGCTGGTCTGAGAGGATGATCAGCCACACTGGGACTGAGACACGGCCCAGACTCCTACGGGAGGCAGCAGTGGGGAATATTGGACAATGGGCGCAAGCCTGATCCAGCAATGCCGCGTGTGTGAAGAAGGTTTTCGGATTGTAAAGCACTTTCAGCGGGGACGATGATGACGGTACCCGCAGAAGAAGCCCCGGCTAACTTCGTGCCAGCAGCCGCGGTAATACGAAGGGGGCAAGCGTTGCTCGGAATGACTGGGCGTAAAGGGCGCGTAGGCGGTTGTTACAGTCAGATGTGAAATTCCCGGGCTTAACCTGGGGGCTGCATTTGATACGTGATGACTAGAGTGTGAGAGAGGGTTGTGGAATTCCCAGTGTAGAGGTGAAATTCGTAGATATTGGGAAGAACACCGGTGGCGAAGGCGGCAACCTGGCTCATGACTGACGCTGAGGCGCGAAAGCGTGGGGAGCAAACAGGATTAGATACCCTGGTAGTCCACGCTGTAAACGATGTGTGCTGGATGTTGGGTGACTTTGTCATTCAGTGTCGTAGTTAACGCGATAAGCACACCGCCTGGGGAGTACGGCCGCAAGGTTGAAACTCAAAGGAATTGACGGGGGCCCGCACAAGCGGTGGAGCATGTGGTTTAATTCGAAGCAACGCGCAGAACCTTACCAGGGCTTGACATGCGGAGGCTGTGTCCAGAGATGGGCATTTCTCGCAAGAGACCTCCAGCACAGGTGCTGCATGGCTGTCGTCAGCTCGTGTCGTGAGATGTTGGGTTAAGTCCCGCAACGAGCGCAACCCTCGCCTTTAGTTGCCATCACGTCTGGGTGGGCACTCTAAAGGAACTGCCGGTGACAAGCCGGAGGAAGGTGGGGATGACGTCAAGTCCTCATGGCCCTTATGTCCTGGGCTACACACGTGCTACAATGGCGGTGACAGTGGGAAGCCAGGTGGTGACACCGAGCCGATCTCAAAAAGCCGTCTCAGTTCGGATTGCACTCTGCAACTCGAGTGCATGAAGGTGGAATCGCTAGTAATCGCGGATCAGCATGCCGCGGTGAATACGTTCCCGGGCCTTGTACACACCGCCCGTCACACCATGGGAGTTGGTTTGACCTTAAGCCG
