# Supplementary material for: Metabolic Investigation in Gluconacetobacter xylinus and Its Bacterial Cellulose Production under a Direct Current Electric Field
Source: Front Microbiol. 2016 Mar 17;7:331. doi: 10.3389/fmicb.2016.00331 (PMC4794480; doi:10.3389/fmicb.2016.00331)
Supplement: Supplementary file 4 [file DataSheet4.DOCX]

Fig. S4 Changes of amino acids in G*. xylinus* cultured with/without DC electric field
